# Lexical Planning in People Who Stutter: A Defect in Lexical Encoding or the Planning Scope?

**DOI:** 10.3389/fpsyg.2021.581304

**Published:** 2021-02-23

**Authors:** Liming Zhao, Miaoqing Lian

**Affiliations:** ^1^Key Research Base of Humanities and Social Sciences of the Ministry of Education, Academy of Psychology and Behavior, Tianjin Normal University, Tianjin, China; ^2^Faculty of Psychology, Tianjin Normal University, Tianjin, China; ^3^Center of Collaborative Innovation for Assessment and Promotion of Mental Health, Tianjin, China

**Keywords:** stuttering, EXPLAN model, lexical planning scope, speech production, lexical defects

## Abstract

Developmental stuttering is a widely discussed speech fluency disorder. Research on its mechanism has focused on an atypical interface between the planning (PLAN) and execution (EX) processes, known collectively as the EXPLAN model. However, it remains unclear how this atypical interface influences people who stutter. A straightforward assumption is that stuttering speakers adopt a smaller scope of speech planning, whereas a defect in word retrieval can be confounding. To shed light on this issue, we took the semantic blocking effect as an index to examine lexical planning in word and phrase production. In Experiment 1, for word production, pictures from the same semantic category were combined to form homogeneous blocks, and pictures from different categories were combined to form heterogeneous blocks. A typical effect of semantic blocking showing longer naming latencies for homogeneous blocks than heterogeneous ones was observed for both stuttering and fluent speakers. However, this effect was smaller for stuttering speakers, when it was subject to lexical defects in stuttering. In Experiment 2, for a conjoined noun phrase production task, the pictures referring to the first noun were manipulated into homogeneous and heterogeneous conditions. The semantic blocking effect was also much smaller for stuttering speakers, indicating a smaller scope of lexical planning. Therefore, the results provided more evidence in support of the EXPLAN model and indicated that a smaller scope of lexical planning rather than lexical defects causes the atypical interface for stuttering. Moreover, a comparison between these two tasks showed that the study findings have implications for syntactic defects in stuttering.

## Introduction

Speech communication is one of the most complex cognitive-motor activities that humans engage in. Even simple speech requires the orchestration of high-level cognition, in the form of the intended message. These messages are mapped to corresponding lexical and phonological representations, which are then expressed by precisely timed muscle contractions of the vocal articulators (Levelt, 1989; Indefrey and Levelt, 2004). This complexity provides many opportunities for speech to fail, which is evident in developmental stuttering and other speech disorders (Mock et al., 2016). Developmental stuttering originates in childhood and is reflected by sound and syllable repetitions, prolongations, and silent blocks that disrupt the natural flow of speech (Bloodstein and Bernstein Ranter, 2008). People can generally recover from developmental stuttering without any intervention, but research has indicated that at age 12 and older, the severity ratings of recovered speakers and their dysfluency counts drop (Howell et al., 2008).

The general consensus is that language planning and speech motor programming and execution are affected in people who stutter. For example, in their covert repair hypothesis (CRH), Kolk and Postma (1997) proposed that all disfluent speech is caused by “covert repairs” of phonological encoding errors that speakers detect before they are expressed overtly. Moreover, some research has revealed that adults who stutter (AWS) have difficulty in phonological processing (Weber-Fox, 2001; Weber-Fox et al., 2004; Sasisekaran et al., 2006). However, the CRH does not adequately account for childhood stuttering. Although some early studies showed that the stuttering frequency of young stutterers may increase with phonological complexity (longer words, for example) (Schlesinger et al., 1966), more recent studies showed that speech fluency in children who stuttered did not change systematically with the increasing phonological complexity of words and non-words (Hakim and Ratner, 2004; Seery et al., 2006; Buhr et al., 2016). Thus, Savage and Howell (2008) developed the EXPLAN model, which proposes that the planning defects are global and not specifically phonological.

**Figure 1:**
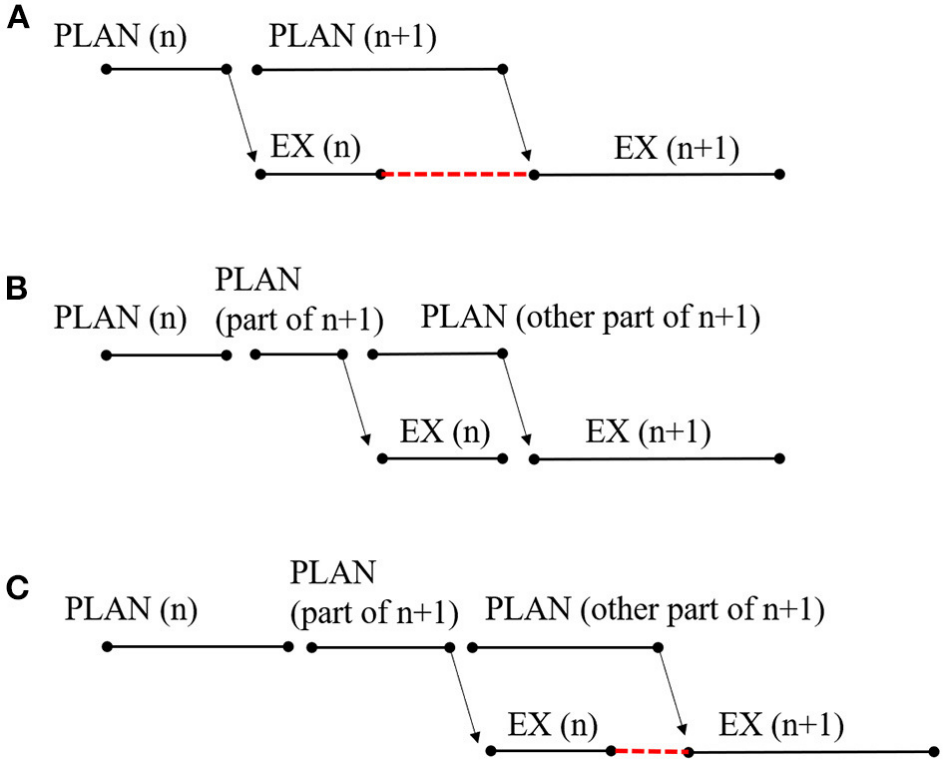
Illustration of **(A)** original EXPLAN model, **(B)** fluent speech according to previous study of planning scope, and **(C)** assumption of lexical defect for stuttering.

The EXPLAN model proposes that there is an atypical interface between planning and execution processes (Howell, 2002, 2004). That is, failure in speech fluency may result from mismatching between the cognitive–linguistic formulation of a speech plan and the motor execution of the linguistic plan (Howell, 2007a, 2010; Lu et al., 2010). The name “EXPLAN” was derived from “EX,” the speech-execution mechanism, and “PLAN,” the parallel language planning mechanism (Howell, 2004; Savage and Howell, 2008). According to the EXPLAN model, the contexts in which fluency is likely to fail are when EX(n) is short and PLAN(n + 1) is long (Howell and Au-Yeung, 2002). Here, we created a similar figure to Howell (2010) to represent the temporal relationship between planning and execution diagrammatically when a speaker stutters (Figure 1A). When planning and executing two successive words (the words n and n + 1), the execution of word n commences shortly after it is planned. When execution of word n is complete, the plan for word n + 1 is not finished. A gap between the execution of word n and n + 1 will occur (represented as the red line of dashes) if speakers do not say anything, inducing a silent block that is considered as a type of stuttering. Speakers may repeat or prolong the articulation of the whole word n or advance part of the word n + 1 to fill this gap, thus inducing other types of stuttering. Take the utterance “I split it” as an example. The speaker starts by planning “I,” which is a simple and short word. Once the plan is ready, it can be sent to the motor execution processes and articulated rapidly. While “I” is articulated, planning for the verb “split” can be performed, which is harder and takes more time to plan than the word “I.” Thus, when “I” has been uttered, only the word “I” and part of the word “split” have been planned and are ready for execution, resulting in disfluencies like “I I split it,” “I [pause] split it,” “I sssplit it,” and so on.

The diagrammatic framework of the EXPLAN theory provides a plausible explanation for disfluent speech. However, it seems that the tendency to make speech errors, such as stuttering, especially when uttering words that are difficult to plan and follow a short word, is the same for stuttering and fluent speakers. This idea of one mechanism resulting in all forms of disfluent speech, including “normal disfluencies” and “stuttered speech,” creates a problem: What differs between stuttering and fluent speakers? In fact, speech errors are rare for fluent speakers, whereas people of all ages who are considered stutterers make many more speech errors than their age-matched fluent speakers. The reason for this, according to the EXPLAN model, remains unclear. In other words, it is urgent that we explain why stuttering speakers face a mismatch between planning and execution more frequently than fluent speakers. This issue was explored in the current study.

One straightforward possibility is that compared to stuttering speakers, fluent speakers may plan more information ahead before initiating speech. Let us return to the diagrammatical illustration of Figure 1A. The red dash line represents a time gap between the execution of word n and n + 1, which appears as the execution of word n commences, shortly after its plan. However, let us say that the speaker plans the word n plus part of information of word n + 1 before the execution of word n. Then, although it will take longer for speakers to initiate the speech, there will be enough time to plan for the left part of word n + 1 while executing word n, and the speech will be fluent without time gaps (see Figure 1B). This parameter of how far ahead speakers plan before they utter speech, referred to as the scope of planning, has been a central issue for the past few decades (Zhao et al., 2015). There has been little research on the planning scope of children or stutterers. However, many studies showed that for fluent adults, when producing a long utterance, such as a phrase or a sentence, speakers could plan more than one word before speech onset (Meyer, 1996; Allum and Wheeldon, 2007, 2009; Zhao et al., 2015, but see Griffin, 2001; Zhao and Yang, 2016).

In many studies, the planning scope in sentence production was investigated by asking participants to verbally describe the relationship between two or more distinct objects and then comparing onset latencies across utterance formats. For example, Allum and Wheeldon (2007) compared onset latencies between sentences with a prepositional phrase modifying the subject (e.g., “The dog above the flower is red”) and sentences with a conjoined noun phrase as the subject (e.g., “The dog and the flower are red”). They observed slower onset latencies for conjoined utterances than for prepositional utterances. As these two types of utterances have the same length for the subject and for the whole sentence, Allum and Wheeldon concluded that speakers adopt the first functional phrase as the planning scope in conjoined utterances (e.g., “the dog and the flower”) and in prepositional utterances (e.g., only “the dog”). Even more closely related to the issue of the current study, Griffin (2003) found that when speakers were asked to name two objects successively, such as “wig, carrot” or “windmill, carrot,” it took longer for them to initiate the utterance when the first word was monosyllabic (e.g., wig) than multisyllabic (e.g., windmill). As multisyllabic words take more time to prepare than the monosyllabic ones, this reversed word length effect showed that speakers spent more time preparing the second word when the first one was short. In other words, the planning scope is larger when the first word is short. This fits the illustration in Figure 1B, which shows that fluent adult speakers planned ahead for some of the information in word n + 1 before speech onset when the first word was short.

It is worthy to note that the aforementioned studies concerned the planning scope in a general way. However, the planning scope can be assessed for each of the processing levels involved in speech planning. As mentioned earlier, there are at least two processing steps for retrieving the corresponding lexical and phonological information (Levelt, 1989; Indefrey and Levelt, 2004). The planning scope involved at these two levels has been investigated in increasing detail as research has deepened. The focus of the present study was primarily on the issue of lexical planning scope.

The lexical planning scope in speech production, that is, how far ahead speakers plan lexically before they start producing an utterance, has primarily been investigated using classical paradigms for word production, such as the picture–word interference paradigm and the blocked-cyclic naming paradigm. Meyer (1996) picture–word interference experiments showed a semantic interference effect in the onset latencies for the second noun of phrases (e.g., “the dog and the flower”) and sentences (e.g., “The dog is next to the flower”). The semantic interference effect in onset latencies, referring to the effect of longer onset latencies when a distractor is categorically related (e.g., “dog”) to the target word (e.g., “cat”) rather than unrelated (e.g., “door”), often poses difficulty in lexical selection during word production (Levelt et al., 1999). Therefore, Meyer interpreted her results as evidence of the clausal scope of lexical planning. Using the same logic, Zhao and Yang (2016) used the semantic blocking effect in the blocked-cyclic naming paradigm as an index to investigate lexical planning scope. In the classic paradigm of blocked-cyclic naming, participants are asked to name a small set of pictures one by one, which are presented repeatedly within a block (Damian et al., 2001; Belke et al., 2005). The semantic blocking effect in onset latencies refers to the effect of longer onset latencies existing when all the to-be-named items in the block represent the same semantic category (e.g., “dog,” “cat,” and “zebra” from animals, called a homogeneous block) than when they represent different categories (e.g., “dog,” “chair,” and “grapes” from animals, furniture, and fruit, respectively, called a heterogeneous block). As this latency effect is also considered to pose difficulty in lexical selection (Kroll and Stewart, 1994; Howard et al., 2006; Navarrete et al., 2010), a significant latency effect of semantic blocking is expected on words that are lexically selected before speech onset when producing multiple word utterances. Zhao and Yang (2016) asked participants to produce a sentence with a conjoined noun phrase as the subject, such as “The chair and the boat are both red.” In Experiment 1, they manipulated the first noun (e.g., “chair”) into homogeneous and heterogeneous blocks and found a semantic blocking effect in the onset latencies, indicating that the first noun is lexically selected before speech onset. However, in Experiment 2, they manipulated the second noun (e.g., “boat”) into homogeneous and heterogeneous blocks and found no such effect in the onset latencies. They thus concluded that the lexical planning scope for fluent adult speakers does not encompass this second noun phrase and discussed the discrepancies between their findings and Meyer's conclusion.

The exact scope of lexical planning remains a controversial issue. We did not delve into this controversy in this study, especially considering that the lexical planning scope may vary according to the cognitive load and other linguistic factors of speech (Wagner et al., 2010; Wheeldon et al., 2013). Instead, this study aimed to test whether children who stutter (CWS) adopt a smaller scope of lexical planning than children who do not stutter (CWNS), resulting in stuttering in childhood. Here, we used the same method as Zhao and Yang (2016) to test and compare the lexical planning scope of CWS and CWNS when producing a conjoined noun phrase (e.g., “the chair and the boat”). As mentioned above, Zhao and Yang (2016) found that only the first noun of the conjoined noun phrase was lexically selected before speech onset. Therefore, the current study only adopted Experiment 1 of Zhao and Yang (2016) for efficiency.

In the current study, participants were asked to produce a conjoined noun phrase of “N1 and N2” (no determiners in Mandarin) corresponding to two pictures vertically presented at the same time in each trial. As the Experiment 1 of Zhao and Yang (2016) showed, the first noun (the top picture) of each utterance was manipulated to be either homogeneous or heterogeneous in each block of trials, whereas the second noun was always heterogeneous in all blocks. We expected that for the CWNS, there would be a semantic blocking effect in onset latencies as the fluent adult speakers demonstrated in Zhao and Yang (2016) Experiment 1. If the CWS adopted a smaller scope of lexical planning, they would not lexically retrieve the first noun of the utterance as completely as the CWNS would before speech onset. As a result, a smaller or even no semantic blocking effect in onset latencies would be observed for the CWS. Most previous studies have not reported on the semantic blocking effect in terms of error rates because these rates are too low in fluent adult speakers (Zhao and Yang, 2016). However, we expected that this effect might appear in the children in the current study, as speech ability continues to develop until adulthood (Swanson and Howell, 2001; Kronenberger et al., 2013), and thus children may make more speech errors than adults. In addition, CWS tend to make more disfluent speech, which is also considered as a kind of speech error (Pellowski and Conture, 2005), so we expected them to have higher error rates than the CWNS regardless of homogeneous or heterogeneous blocks. However, the magnitude of the semantic blocking effect in error rates between the CWS and the CWNS needs further discussion.

Although the semantic blocking effect is often interpreted as a source of difficulty in lexical selection in both onset latencies and error rates, it does not always coincide with these two parameters. For example, if we manipulated the second noun of the conjoined noun phrase into homogeneous and heterogeneous blocks, and the speakers did not lexically select the second noun before speech onset, there would be no semantic blocking effect in the onset latencies. However, speakers must lexically select the second noun before producing it, and thus they make more errors, such as hesitating between the execution of the first and second nouns or selecting the wrong word, when the second noun is more difficult to select in the homogeneous blocks. In short, the semantic blocking effect in error rates mainly reflects the difficulty of lexical selection itself rather than the planning scope. As in the current study, we expected the first noun to be more difficult to retrieve lexically in homogeneous blocks than in heterogeneous ones. Therefore, a semantic blocking effect may also be observed in error rates, even though there would be a smaller or even no semantic blocking effect in onset latencies for the CWS. In addition, the magnitude of the semantic blocking effect in error rates between the CWS and the CWNS reflects whether it would be more difficult for the CWS to lexically select words than the CWNS. However, it is not easy to speculate at the result of such an experiment, as the issue of whether a person who stutters has difficulty with lexical selection remains controversial (Weber-Fox, 2001; Pellowski and Conture, 2005; Hartfield and Conture, 2006; Hennessey et al., 2008). More importantly, the defects in lexical selection may confound the assumption that a smaller planning scope is the cause of stuttering.

As shown in Figure 1C, if the CWS adopted the same scope of lexical planning as the CWNS but had defects in lexical selection, it would take more time for them to plan lexical information before speech onset, inducing longer onset latencies. Moreover, compared to Figure 1A, it would also take more time for the CWS to lexically plan for the other part of word n + 1 after speech onset, but the execution of word n would remain consistent as in fluent speech. As a result, there would be still a time gap between the execution of word n and n + 1. Thus, before attributing stuttering to smaller scope of lexical planning, we had to test whether the CWS had difficulty with lexical encoding. Comparing the magnitude of the semantic blocking effect in error rates between the CWS and the CWNS could provide information for this test. If the CWS did have lexical defects, the semantic blocking effect in error rates would be larger for the CWS than for the CWNS. Otherwise, their semantic blocking effect in error rates would be equivalent. However, this method would be risky, because the semantic blocking effect in error rates may be absent as it is in adult speakers.

The classic way to test the lexical defects in CWS also takes the semantic effect in the word production as the index. Pellowski and Conture (2005) conducted a picture–word interference study with word production task, in which a word semantically related or unrelated to the target picture was presented auditorily 700 ms before displaying a picture. They found a semantic priming effect in the naming latencies for CWNS aged 3–6 years old, but for the CWS, there was a semantic interference effect in latencies. These results suggested that CWS have more difficulty with lexical encoding. Unfortunately, the semantic effect in error rates was not reported in this study, though it may strengthen the conclusion of lexical defects with larger semantic interference for the CWS or lead to a discussion of the speed–accuracy trade-off. However, Hennessey et al. (2008) conducted a similar study of adults who stuttered and found no significant difference in the magnitude of semantic interference effect between those who stuttered and normally fluent speakers. This divergence may stem from the fact that most CWS spontaneously recover by adulthood (Andrews et al., 1983; Yairi and Ambrose, 1999). As the severity ratings of the recovered speakers dropped dramatically at age 12 and beyond (Howell, 2007b; Howell et al., 2008), we recruited participants in this age range and used the blocked-cyclic naming paradigm in word production to test lexical defects in order to help fill the age gap of this issue and solve the debate on persistent stutterers.

The word production experiment (Experiment 1) was to test whether CWS at age 12 and up have defects in lexical encoding. All of the pictures used in the phrase production experiment (Experiment 2) to test the planning scope were manipulated to be either homogeneous or heterogeneous in each block of trials. Participants were then asked to name a single picture one by one with a single noun. Under the same logic of previous tests using the picture–word interference paradigm, we expected that if CWS had more difficulty to lexically select the target word, a larger semantic blocking effect in naming latencies would be observed for CWS than for CWNS. This expectation stemmed from the assumption that speakers must complete lexical selection before a single word is produced (Levelt, 1989). However, whether this would be true for the CWS remained a question. If the CWS adopted a lexical planning scope smaller than the first noun as we expected in the phrase production task, they might not fully complete the lexical selection before the single word production either. Thus, the results of the word production task were expected to have exactly the same pattern as the ones of the phrase production task.

## Experiment 1

A blocked-cyclic naming task with single-word production was conducted to test whether the CWS at age 12 and beyond have defects in lexical selection.

### Method

#### Participants

Participants consisted of 22 CWS and 24 CWNS from a middle school, all of whom were native Chinese speakers. Participants were between the ages of 12 and 13 years old (CWS: *M* = 12.3, *SD* = 0.5; CWNS: *M* = 12.4, *SD* = 0.5) with no statistically significant between-group difference [*t*_(44)_ = −0.40, *p* = 0.69] in chronological age. The CWS group consisted of 15 boys and seven girls, and the CWNS group consisted of 17 boys and seven girls. None of the 22 children had received formal/structured intervention for stuttering or any other communication disorder prior to participation in this study. Also, participants had no known or reported hearing, neurological, developmental, academic, intellectual, or emotional problems. This study's protocol was approved by the Institutional Review Board at Tianjin Normal University. For each of the 46 participants, parents signed an informed consent form, and their children assented.

In order to avoid the label of stuttering on these children and any other negative influence potentially induced by this label, we used the following procedure to recruit participants. First, we asked all of the children (more than 300) between the ages of 12 and 13 years old in that middle school to self-assess the revised version by Van Zaalen et al. (2009) of the questionnaire of Predictive Cluttering Inventory (PCI). According to previous data of PCI compared with clinical experience with stuttering and cluttering (another fluency disorder that has similar symptoms as stuttering) clients, a total score between 80 and 120 was considered as potential stuttering, whereas a total score above 120 points was sufficiently able to detect possible cluttering components in speech (Daly and Cantrell, 2006; Van Zaalen et al., 2009). Thus, we contacted the parents whose children got a PCI score between 80 and 120. Twenty-two of them reported stuttering onset before the age of 5 and were willing to participate further studies. Similarly, we recruited another 24 children whose score of PCI was <60, and their parents reported no stuttering and were also willing to participate further studies. Then, the children's speech samples were collected and analyzed by at least two speech-language pathologists based on the Stuttering Severity Instrument-4 (SSI-4; Riley, 2009). No scores or results were fed back to the children, but their parents could obtain the results if they desired.

#### Classification

CWS—A child was considered a CWS if he or she (a) self-assessed the revised version PCI and received a total score between 80 and 120 (CWS had a mean score of 98.82, *SD* = 14.03) and (b) had a total score of 12 or above (a severity equivalent of at least “mild”) on the SSI-4 (CWS had a mean score of 18.82, *SD* = 3.47).

CWNS—A child was considered a CWNS if he or she (a) self-assessed the revised version PCI and received a total score <60 (CWNS had a mean score of 34.04, *SD* = 16.39) and (b) had an overall score of 11 or less (a severity equivalent of less than “mild”) on the SSI-4 (CWNS had a mean score of 9.33, *SD* = 1.13).

#### Materials

Eighteen pictures were selected from the picture pool of Snodgrass and Vanderwart (1980). They were divided into six semantic categories, each consisting of three items (see Appendix), which were the same as those used by Zhao and Yang (2016). Three of these categories (zoo animals, fruits, and furniture) were manipulated into homogeneous and heterogeneous blocks independently from the other three categories (transports, musical instruments, and body parts). For a future comparison between Experiments 1 and 2, we called the former three categories as top ones, and the other three categories as lower ones. That is, for the three top categories, items from the same category were combined to form three homogeneous blocks (i.e., “gorilla,” “elephant,” and “zebra” from the zoo animals present within one block; “apple,” “banana,” and “grapes” from the fruits present within one block; and “dresser,” “chair,” and “couch” from the furniture present within one block). One picture at a time was extracted from these three categories to form three heterogeneous blocks (i.e., “gorilla,” “apple,” and “dresser” present within one block; “elephant,” “grapes,” and “couch” present within one block; and “zebra,” “banana,” and “chair” present within one block). Every three target items within each block did not have a phonological relationship. Within each block, each of the three target pictures was presented six times in a pseudorandom order, such that the same picture never appeared in consecutive trials. In these settings, items in the homogeneous blocks were exactly the same as in the heterogeneous ones and presented the same number of times. Therefore, if there was any effect caused by phonological complexity, it would be counterbalanced when comparing the homogeneous and heterogeneous blocks, leaving only the lexical/semantic effect. The other three categories were used in the same way to form another three homogeneous and three heterogeneous blocks. Six filler items were presented for practice, and they were not from the experimental categories.

#### Design

Stuttering (CWS vs. CWNS) was a between-participant factor, whereas Blocking (homogeneous vs. heterogeneous) was manipulated as a within-participant factor. Twelve experimental blocks (six homogeneous and six heterogeneous blocks) were presented in an AABB design. For the first three trials in each block, each of the three target pictures was presented once. For the rest of the trials in the block, each of the target pictures was presented five times in a pseudorandom order, such that the same picture never appeared in consecutive trials.

#### Procedure

Participants were seated in front of the computer screen, at a distance of about 60 cm, and tested individually. A fixation point appeared on the screen for 600 ms. Then, the target picture appeared for 2,000 ms. Participants were asked to name the picture as accurately and quickly as possible. There was a blank interval of 1,000 ms between consecutive trials. Before starting each block, the three pictures used in this block were presented successively in a random order and accompanied by their names so that participants could get familiar with them. The screen's background remained black. The whole testing session lasted about 15 min, and participants had a short break between blocks.

Response latencies were measured from the stimulus onset to the speech onset, which was triggered by the vocal sound through a microphone connected to the computer. Stimulus presentation, reaction times, and response recording were controlled by the program DMDX (Forster and Forster, 2003). The speech errors were recorded online by the experimenter, and re-checked offline by another researcher according to the recording of the voice response.

### Results

In this and a subsequent experiment, we analyzed the correct RT and error rates for the fixed factors of Stuttering and Blocking, using the linear mixed-effects model with participants and items as crossed random factors (Baayen, 2008). The dependent variables were speech onset latency and error rate. The speech onset latency was defined as the time that elapsed from the onset of the display to articulation of the first word of the target utterance. In this experiment, the target utterance was the single word referring to the target picture and was considered as the item in the linear mixed-effects model.

Of the 216 experimental trials for each participant, recording failures and the data points faster than 200 ms were excluded from the analyses. Then, data points more than three standard deviations from the mean response latency of the respective participant and semantic context condition were excluded as outliers. All the excluded trials accounted for 8.7% of the data.

Production errors were scored as fluency problems (revisions, repetitions, prolongation, interjections, and broken words) and wrong names. Such trials accounted for 3.0% of the data and were excluded from the correct RT analyses. Finally, 8,781 trials were left for the correct RTs analysis. The mean correct RT and error rates are summarized in Table 1.

**Table 1:** Mean latencies and percentage error rates for two experiments (standard deviation in brackets).

**Tasks**	**Blocking**	**CWS**		**CWNS**	
		**Correct RT (ms)**	**Error rates (%)**	**Correct RT (ms)**	**Error rates (%)**
Word production (all items)	Homo	714 (288)	5.7 (23.2)	811 (307)	3.0 (17.2)
	Hetero	661 (268)	3.1 (17.3)	658 (277)	1.3 (11.2)
Word production (only N1)	Homo	716 (293)	5.9 (23.6)	815 (297)	4.3 (20.3)
	Hetero	659 (265)	2.6 (16.0)	670 (281)	1.8 (13.4)
Phrase production	Homo	1,014 (457)	8.7 (28.2)	1,057 (465)	5.3 (22.3)
	Hetero	977 (463)	4.8 (21.3)	933 (438)	2.7 (16.3)

### Correct RT

The data were submitted to a linear mixed-effects model using the lme4 package (Bates et al., 2013, Version 1.1-5) implemented in R 3.0.3 (R Core Team, 2014). The *p*-values were estimated using the lmerTest package (Kuznetsova et al., 2013, Version 2.0-11). The mixed-effects results (see Table 2) showed a significant main effect of Blocking, showed no main effect of Stuttering, and revealed a significant interaction between Stuttering and Blocking.

**Table 2:** The model's estimate, standard error (SE), *t* or *Z* value, and *p*-value of fixed effects for the correct RT and error rates in Experiment 1 (with all items).

**Measure**	**Model type**	**Effect**	**Estimate**	***SE***	***t***	***p***
Correct RT	Interactive	(Intercept)	655.48	15.58	42.07	<0.001
		Stuttering × Blocking	−97.86	11.89	−8.23	<0.001
	Simple effects	CWS: semantic effect	55.27	8.66	6.38	<0.001
		CWNS: semantic effect	153.13	8.15	18.80	<0.001
		Heterogeneous blocks: CWS vs. CWNS	1.84	22.41	0.08	0.935
**Measure**	**Model type**	**Effect**	**Estimate**	***SE***	***Z***	***p***
Error rates	Interactive	(Intercept)	−4.55	0.24	−18.63	<0.001
		Stuttering × Blocking	−0.24	0.27	−0.91	0.362
	Main effects	Stuttering	0.74	0.12	6.04	<0.001
		Blocking	0.74	0.13	5.93	<0.001

The model testing the simple effect of Blocking for CWS and CWNS showed that both CWS and CWNS had semantic blocking effect, which refers to longer speech onset latencies in homogeneous blocks than in the heterogeneous blocks. However, the semantic effect was much larger for CWNS than for CWS. For experimental purposes, we also tested the simple effect of Stuttering on heterogeneous blocks. The result showed that there was no statistical difference between the CWS and the CWNS in the onset latency for heterogeneous conditions.

### Error Rate

The error data were analyzed using a logit mixed model (Jaeger, 2008) using the same model as for correct RT [Error rates ~ Stuttering ^*^ Blocking + (1 | participant) + (1 | item)]. The model (see Table 2) showed significant main effects of Blocking and Stuttering, but no significant interaction between them. The *post-hoc* test model for Stuttering [Error rates ~ Stuttering + (1 | participant) + (1 | item)] showed that error rates were significantly larger for CWS than for CWNS, whereas the model for Blocking showed that there was a significant effect of semantic blocking, which refers to larger error rates in the homogeneous blocks than in the heterogeneous blocks.

### Discussion

**Figure 2:**
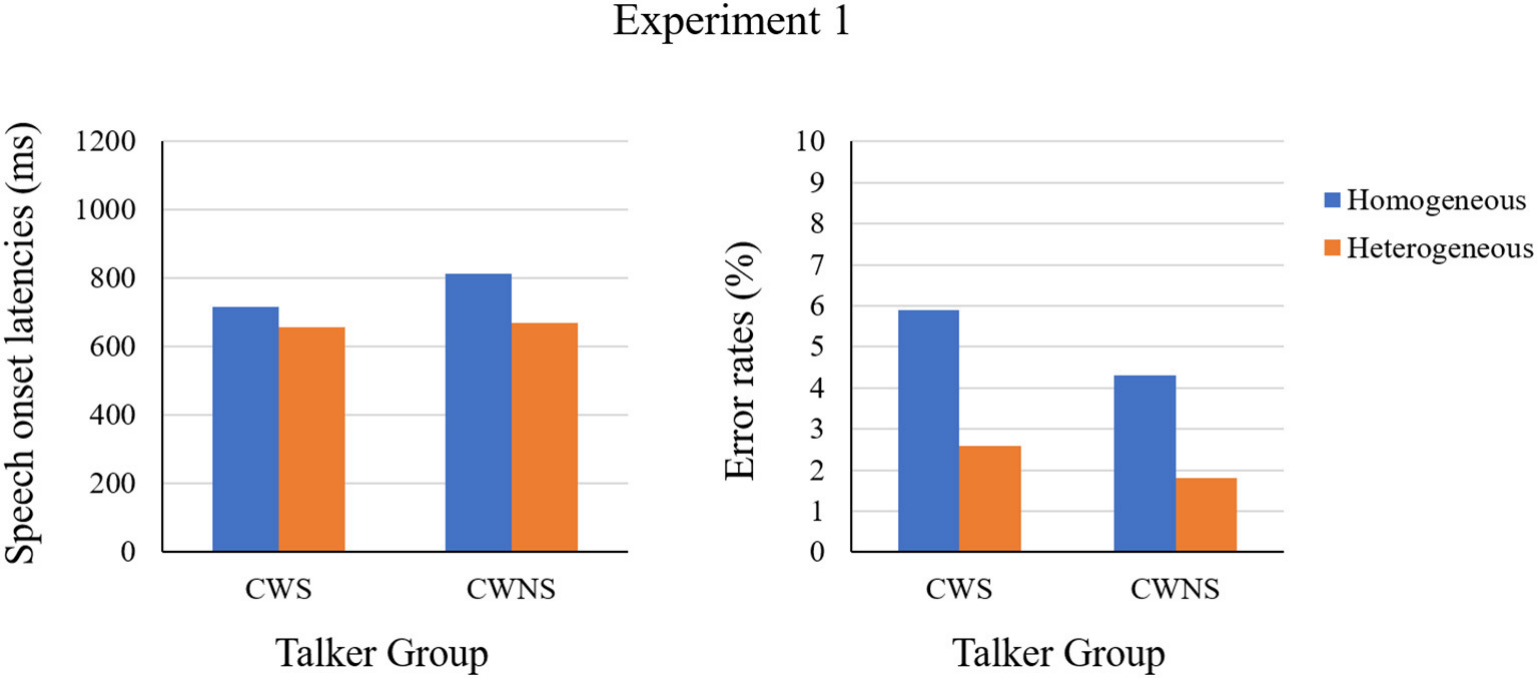
The means of speech onset latencies and error rates grouped by the CWS and the CWNS in Experiment 1.

**Figure 3:**
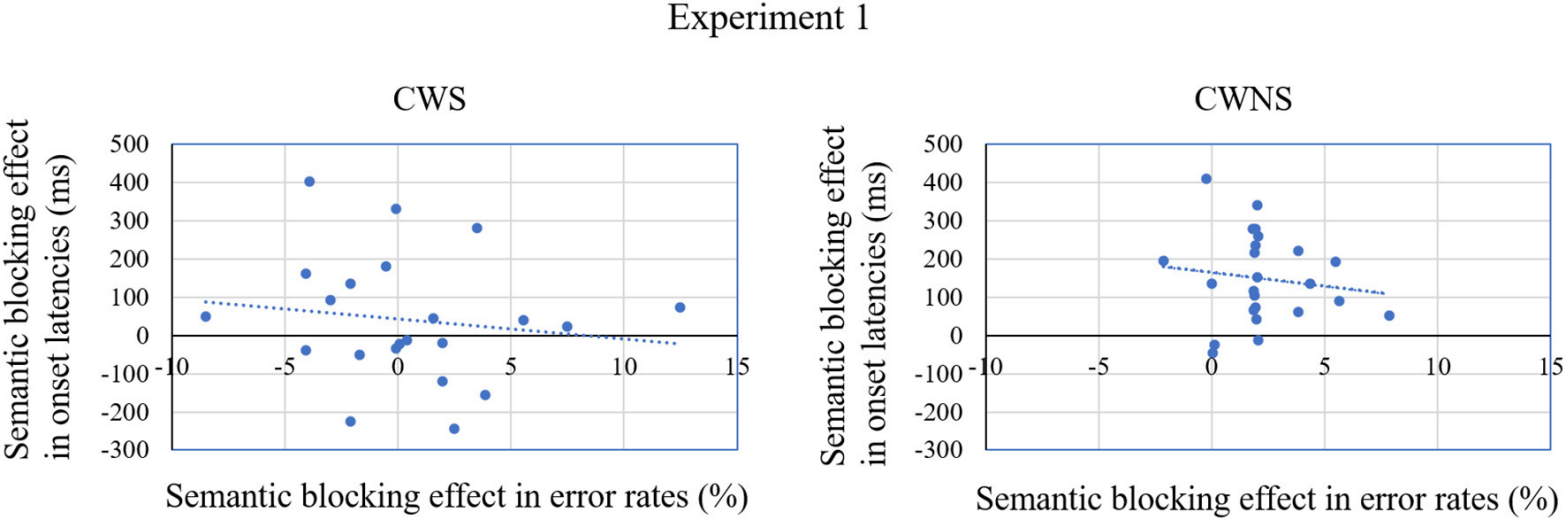
The individual effects of semantic blocking in speech onset latencies and error rates (Homogeneous blocks – Heterogeneous blocks) grouped by the CWS and the CWNS in Experiment 1.

In Experiment 1, we tested whether the CWS have defects in lexical selection by a word production task in the blocked-cyclic naming paradigm. The results of naming latencies showed a semantic blocking effect for both CWS and CWNS, which was consistent with previous studies of blocked-cyclic naming paradigm (Damian et al., 2001; Belke et al., 2005). The results of error rates also showed a semantic blocking effect for both CWS and CWNS. This result helped mitigate the intuitive speculation that the semantic effects in naming latencies are induced by a speed–accuracy trade-off. From the perspective of the statistical mean of the effect, the trends in naming latencies and error rates were the same. That is, for both CWS and CWNS, there were longer naming latencies and more errors in the homogeneous blocks than in the heterogeneous ones (see Figure 2). From the perspective of an individual effect, the relation between the semantic blocking effect in latencies and error rates shown in Figure 3 was almost the same for CWS and CWNS. Thus, if there is any difference in the magnitude of the semantic blocking effect between CWS and CWNS, it is unlikely to be due to a simple trade-off strategy of speed and accuracy.

Moreover, we did not find any evidence to support lexical defects in the CWS. If the CWS had defects in lexical selection, the naming latencies in the heterogeneous blocks would be longer for the CWS than the CWNS. In addition, the semantic blocking effect in onset latencies and/or error rates would be larger for the CWS than for the CWNS. None of these expectations was found in the results of the current experiment. Firstly, we found no statistical difference in the naming latencies between the CWS and the CWNS in the heterogeneous blocks. Secondly, there was no statistical difference in the magnitude of the semantic blocking effect in error rates between CWS and CWNS, and the semantic blocking effect in latencies for the CWS was even smaller than for the CWNS. All these results indicated that there is no defect in lexical selection for the CWS. They were also consistent with those of a previous study on the auditory priming effect in picture naming with adult stutterers, which found no difference in the general naming latencies and priming effects between adult stutterers and matched controls (Hennessey et al., 2008).

More interestingly, the magnitude of the semantic blocking effect in naming latencies was smaller for the CWS than for the CWNS. This result again was the opposite of a lexical defect for CWS. One possible explanation for it is that stuttering speakers pay more attention to speech production (Arends et al., 1988) and thus can more sufficiently overcome competition in lexical selection. However, this was unlikely to be the case in the current study. If stuttering speakers were more sufficient in their lexical selection, there would be fewer errors for them compared to fluent speakers. In fact, consistently and across blocks of semantic context conditions, we found more speech errors for CWS than for CWNS. A finally plausible reason is that CWS adopt a smaller scope of lexical planning than CWNS, even in single word production. Many studies with fluent adult speakers assigned a task requiring participants to respond to a pictured object with a single noun, and they confirmed that all of the processing required for the planning of the entire utterance is complete before articulation is initiated (Meyer et al., 1998; Meyer and van der Meulen, 2000; Griffin, 2001). However, how stuttering speakers (especially during childhood) plan a single word is still unclear. If CWS do not always complete the lexical selection for the upcoming word, they may begin the speech earlier in a situation of high lexical competition, such as the case with homogeneous blocks. That may be the reason why they are more inclined to make speech errors. This speculation was further tested in Experiment 2 with noun phrase production.

## Experiment 2

The semantic blocking effect in the onset latencies of single-picture naming has been consistently observed in the literature and was also observed in Experiment 1. It was interpreted as a reflection of lexical selection. Zhao and Yang (2016) used this effect as a proxy to test the lexical planning scope in sentence production, and they found that for the conjoined noun phrase as the subject of a sentence, the lexical planning scope only contained the first noun. Thus, in Experiment 2, we asked stuttering and fluent speakers to produce conjoined noun phrase, while manipulating the first noun into homogeneous and heterogeneous conditions of blocks to test the lexical planning scope.

### Method

#### Participants

Participants were the same as in Experiment 1.

#### Materials

The target pictures were the same as in Experiment 1. However, two pictures were presented vertically within each display, and participants were asked to produce the conjoined noun phrase as “N1 and N2.” The N1 and N2 had to be changed to the corresponding names of the pictures, and were not phonologically related. Three categories (zoo animals, fruits, and furniture) served as the first noun in the produced utterances and were always presented in the top position (called top groups); the other three categories (transports, musical instruments, and body parts) were used as the second noun and were always presented in the bottom position (called the bottom groups). N1 was manipulated into homogeneous and heterogeneous blocks, and N2 was always in heterogeneous blocks. That is, for the top groups, items from the same category were combined to form three homogeneous sets as in Experiment 1. In contrast, items from three categories were combined to form three heterogeneous sets also as in Experiment 1. For the bottom group, items from different categories were combined to form three heterogeneous sets and then recombined to form another three heterogeneous sets. Each of the six heterogeneous sets for the bottom groups was paired with one set of the top groups to form six blocks (three homogeneous blocks and three heterogeneous blocks basing on the N1). For example, in one homogeneous block, speakers were asked to name “the gorilla and the airplane,” “the elephant and the drum,” and “the zebra and the finger” six times in a pseudorandom order in which the first noun in the block represented one semantic category (i.e., zoo animals), and the second noun was always from a different semantic category. In one heterogeneous block, speakers were asked to name “the gorilla and the car,” “the apple and the guitar,” and “the dresser and the ear” six times, in which the first noun and second noun represented different semantic categories. Therefore, each noun appeared with the same frequency in the homogeneous and heterogeneous blocks. In addition, as the word length of N1 plays an important role in stuttering in the EXPLAN model illustrated in Figure 1, it remained consistent in the current experiment. That is, all of the names referring to the pictures in the top groups were two-syllable words (see Appendix for their pronunciations in Mandarin Chinese).

#### Design

There were two main factors in Experiment 2, Stuttering (CWS vs. CWNS) and Blocking (homogeneous vs. heterogeneous). These were the same as in Experiment 1, except the Blocking was manipulated only for the first noun of the utterances (the top item). The homogeneous and heterogeneous blocks were presented in alternative order, and the order of blocks was rotated across participants.

#### Procedure

The procedure was almost the same as in Experiment 1, except for the duration of each period. A fixation point appeared on the screen for 1,000 ms. Then, the target picture appeared for 4,000 ms. The blank interval between consecutive trials was 2,000 ms. The whole testing session lasted about 15 min, and participants had a short break between blocks.

### Results

The speech onset latency was defined as the time that elapsed from the onset of the display to articulation of the first word (N1) of the target utterance. In this experiment, the target utterance was the conjoined noun phrase, and the whole phrase was considered as the item in the linear mixed-effects model.

The same criteria were used to exclude 6.5% of the trial data as outliers. Production errors were scored as fluency problems (revisions, repetitions, prolongation, interjections, and broken words), wrong names, and wrong syntax. Such trials accounted for 5.0% of the data and were excluded from the correct RT analyses. Finally, 4,399 trials were left for the correct RT analysis. The mean correct RT and error rates are summarized in Table 1.

### Correct RT

The mixed-effects results (see Table 3) showed significant main effects of Blocking and Stuttering, and revealed a significant interaction between Stuttering and Blocking. The model testing the simple effect of Blocking for CWS and CWNS, respectively, showed that only the CWNS had a significant effect of semantic blocking. The semantic blocking effect for the CWS was marginally significant. In addition, the speech onset latencies were significantly longer for the CWS than the CWNS in heterogeneous blocks.

**Table 3:** The model's estimate, standard error (SE), *t* or *Z* value, and *p*-value of fixed effects for the correct RT and error rates in Experiment 2.

**Measure**	**Model type**	**Effect**	**Estimate**	***SE***	***t***	***p***
Correct RT	Interactive	(Intercept)	931.66	15.06	61.87	<0.001
		Stuttering × Blocking	−87.35	27.45	−3.18	<0.01
	Simple effects	CWS: semantic effect	38.63	20.40	1.89	0.059
		CWNS: semantic effect	125.98	19.33	6.52	<0.001
		Heterogeneous blocks: CWS vs. CWNS	44.34	21.17	2.10	<0.05
**Measure**	**Model type**	**Effect**	**Estimate**	***SE***	***Z***	***p***
Error rates	Interactive	(Intercept)	−3.86	0.22	−17.77	<0.001
		Stuttering × Blocking	−0.03	0.28	−0.10	0.92
	Main effects	Stuttering	0.55	0.13	4.16	<0.001
		Blocking	0.85	0.18	4.72	<0.001

### Error Rate

The model (see Table 3) showed significant main effects of Stuttering and Blocking, but no significant interaction between them. The *post-hoc* test model for Stuttering showed that the error rates were significantly higher for CWS than for CWNS, whereas the model for Blocking showed that there was a significant effect of semantic blocking.

### Discussion

Experiment 2 tested whether the CWS adopt a smaller scope of lexical planning than the CWNS in phrase production. First, the same pattern of error rates was observed as in Experiment 1, showing more errors in homogeneous blocks than heterogeneous ones. Additionally, the magnitude of this semantic blocking effect in error rates was statistically equal for the CWS and CWNS. These results indicated that it is more difficult to lexically select words in homogeneous blocks than in heterogeneous ones, and this difficulty is equal for the CWS and the CWNS. That is, there are no lexical defects in CWS. It is not surprising that the CWS made more speech errors than the CWNS, given group classification procedures.

In addition, it took more time for the CWS to initiate the articulation of a phrase than the CWNS in heterogeneous blocks. This was unlikely to be due to word retrieval, because in Experiment 1, we found no statistical difference between CWS and CWNS in the heterogeneous blocks of single word. Compared to single word production, the syntactic processing in phrase production is obviously more complex. Previous studies showed that fluent speakers syntactically planned for conjoined noun phrases before speech onset (Wheeldon et al., 2013; Zhao and Yang, 2016). Thus, the difference between CWS and CWNS in phrase production may be attributed to syntactic encoding. This is not surprising, as many previous studies indicated that stuttering speakers have defects in syntactic processing (e.g., Ratner and Sih, 1987; Kleinow and Smith, 2000; Cuadrado and Weber-Fox, 2003).

**Figure 4:**
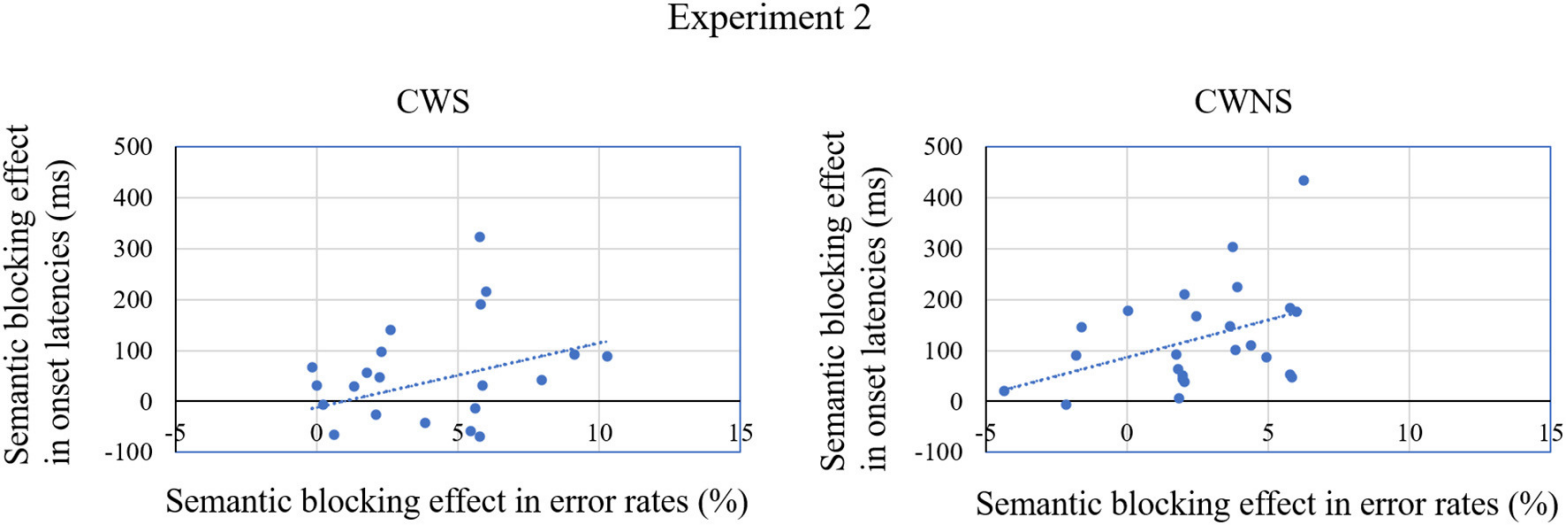
The individual effects of semantic blocking in speech onset latencies and error rates (Homogeneous blocks – Heterogeneous blocks) grouped by the CWS and the CWNS in Experiment 2.

**Figure 5:**
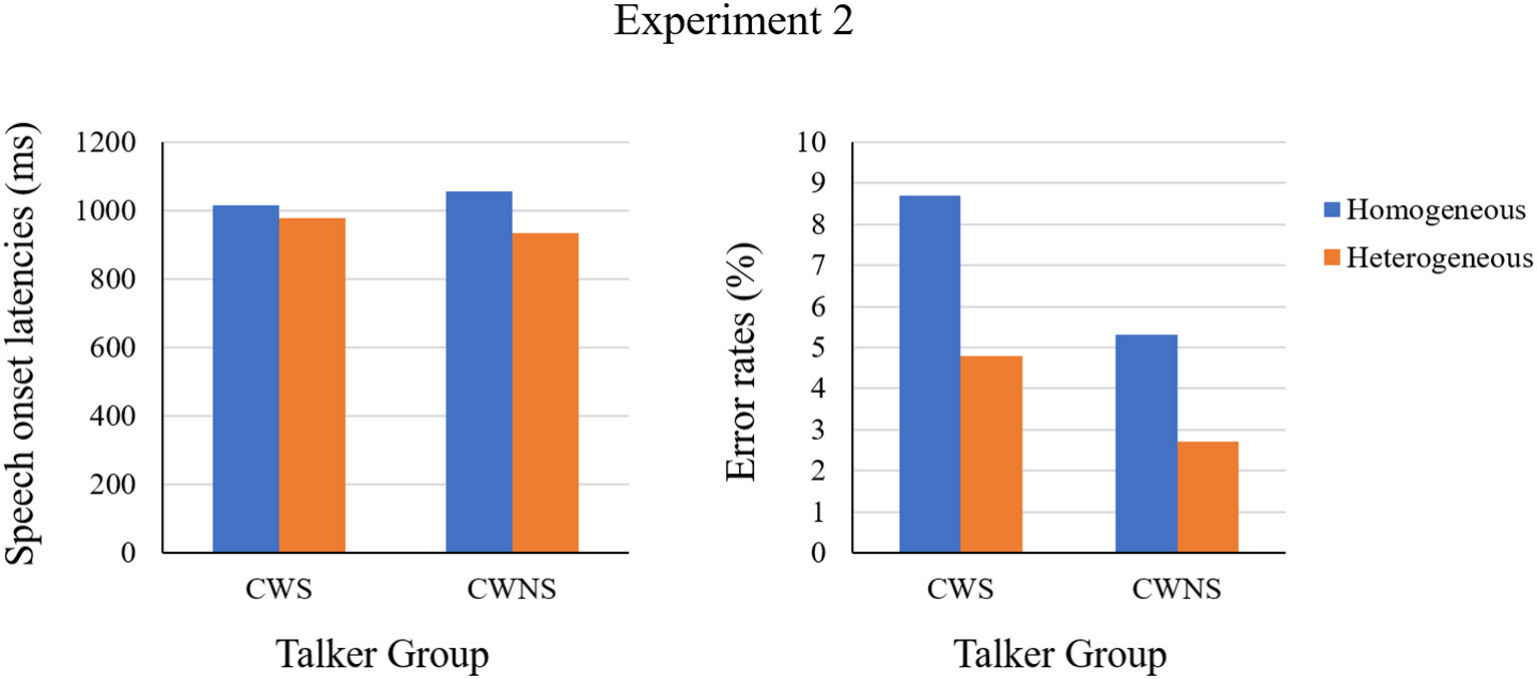
The means of speech onset latencies and error rates grouped by the CWS and the CWNS in Experiment 2.

More importantly, the semantic blocking effect in speech onset latencies for the CWS was much smaller than that for the CWNS, and was even insignificant. This pattern was consistent with the results of Experiment 1, and indicated that CWS adopted a smaller scope of lexical planning than CWNS. In other words, CWS do not constantly complete the lexical selection of the first noun before speech onset, inducing an unstable effect of semantic blocking in speech latencies. As shown in Figure 4, some CWS even exhibited a reversed effect of semantic blocking (longer onset latencies in the heterogeneous blocks than in the homogeneous ones). Figure 4 also shows the relation between the semantic blocking effect in latencies and error rates, which is demonstrated by the slope of the line, and it was quite similar for CWS and CWNS. Moreover, Figure 4 demonstrates that for both CWS and CWNS, the larger the semantic blocking effect was in onset latencies, the larger the effect was in error rates. This was contrary to the explanation of speed and accuracy trade-off. From the perspective of the statistical mean of the effect, the trends in onset latencies and error rates were also the same as in Experiment 1 (see Figure 5). These results provided further evidence to mitigate the strategy of speed–accuracy trade-off as the reason for the smaller effect of semantic blocking in onset latencies for the CWS.

In summary, the pattern of the semantic blocking effect in the onset latencies and error rates of Experiment 1 was the same as that in Experiment 2, with both showing a smaller latency effect for the CWS and a statistically equal effect in error rates between the CWS and the CWNS. This was consistent with the expectations laid out in the *Introduction* and indicated that CWS face more mismatching between planning and execution, which the EXPLAN model proposes is induced by a smaller scope of lexical planning rather than a defect in lexical selection. However, when we considered the results in detail, some minor differences were found between these two experiments. Specifically, the semantic blocking effect in onset latencies for the CWS was significant in Experiment 1 but absent in Experiment 2, and the latency difference between the CWS and the CWNS in heterogeneous blocks was significant in Experiment 2 but absent in Experiment 1. To explain these differences and reinforce our conclusion, we did a comprehensive analysis merging these two experiments. That is, the factor Experiment was added into the linear mixed model used in these two experiments as the third fixed factor. If the pattern of the semantic blocking effect in onset latencies and error rates of Experiment 1 was statistically the same as the one of Experiment 2, there would be no three-way interaction among the factor Experiment and the other two factors (Stutter and Blocking). A main effect of the factor Experiment would be observed in onset latencies and/or error rates, as a noun phrase is more difficult to plan syntactically than a single noun. Moreover, if the latency difference between the CWS and the CWNS in heterogeneous blocks in Experiment 2 was induced by syntactic defects for the CWS, as we discussed earlier, the difference between experiments would be larger for the CWS than for the CWNS. That is, an interaction between the factor Experiment and Stuttering would be observed in onset latencies and/or error rates.

However, before making this analysis, a confounding of between-item variation had to be excluded. In the previous analysis for each experiment separately, the effects of phonological complexity, such as word length and frequency, were counterbalanced by the experimental design of homogeneous and heterogeneous blocks. However, if we merged all of the data of Experiments 1 and 2 in this comprehensive analysis, we could do a comparison of different items when considering the effects of the factor Experiment. In Experiment 1, items from all six categories were manipulated into homogeneous and heterogeneous blocks and analyzed, but only three of them presented as the N1 in the Experiment 2. The data of these three categories in Experiment 1 (half of the data) were picked out, analyzed separately in the following part, and then further compared to the results of Experiment 2. Thus, in the following analysis, the items in Experiment 1 were exactly the same as those of N1 in Experiment 2.

### Comparison of Experiments 1 and 2

Half of the data in Experiment 1 were gathered from three categories of zoo animals, fruits, and furniture, which were presented as the N1 in Experiment 2. There were three homogeneous blocks and three heterogeneous blocks. Using the same criterion, 4,381 trials were left for the correct RT analysis. The mean correct RT and error rates are summarized in Table 1, and the mixed-effects results are shown in Table 4. We can see that the results of model testing with half of the data showed exactly the same pattern as the one with all the items in Experiment 1. These results indicated that the pattern of results observed in Experiment 1 is universal rather than applicable to specific items. The data from Experiment 1 were merged with that of Experiment 2 in the following analysis.

**Table 4:** The model's estimate, standard error (SE), *t* or *Z* value, and *p*-value of fixed effects for the correct RT and error rates in Experiment 1 (with only N1).

**Measure**	**Model type**	**Effect**	**Estimate**	***SE***	***t***	***p***
Correct RT	Interactive	(Intercept)	666.60	17.14	38.90	<0.001
		Stuttering × Blocking	−87.96	16.70	−5.27	<0.001
	Simple effects	CWS: semantic effect	58.50	12.15	4.82	<0.001
		CWNS: semantic effect	146.45	11.46	12.78	<0.001
		Heterogeneous blocks: CWS vs. CWNS	10.76	24.36	0.44	0.660
**Measure**	**Model type**	**Effect**	**Estimate**	***SE***	***Z***	***p***
Error rates	Interactive	(Intercept)	−4.12	0.29	−14.21	<0.001
		Stuttering × Blocking	−0.03	0.35	−0.09	0.932
	Main effects	Stuttering	0.35	0.16	2.19	<0.05
		Blocking	0.87	0.17	5.06	<0.001

A further analysis with three fixed factors of Stuttering (CWS vs. CWNS), Blocking (homogeneous vs. heterogeneous), and Experiment (Experiment 1 vs. 2) was conducted using the linear mixed-effects model with participants and items used as crossed random factors. This analysis was almost the same as that in Experiments 1 and 2, except fixed factor Experiment was added into the model. The results showed no three-way interactions either in speech onset latencies (*b* = −1.85, *SE* = 32.34, *t* = −0.057, *p* = 0.95) or error rates (*b* = 0.002, *SE* = 0.447, *Z* = 0.005, *p* = 0.996). This result, as we expected, excluded the between-item variation and confirmed that the semantic blocking effect between Experiments 1 and 2 was statistically equal. This reinforced the conclusion that CWS adopted a smaller scope of lexical planning in both single-noun naming and noun-phrase production tasks. Meanwhile, a main effect of the factor Experiment was observed significantly in both onset latencies (*b* = −278.88, *SE* = 8.15, *t* = −34.20, *p* < 0.001) and error rates (*b* = −0.389, *SE* = 0.102, *Z* = −3.80, *p* < 0.001), showing longer onset latencies and more errors in the phrase production task than the single-noun production task. This is consistent with the idea that the noun phrase is more difficult to plan syntactically than the bare noun. In addition, the interaction between the factor Experiment and Stuttering was significant in onset latencies (*b* = −56.90, *SE* = 16.32, *t* = −3.49, *p* < 0.001) and not in error rates (*b* = 0.201, *SE* = 0.207, *Z* = −0.970, *p* = 0.332). That is, the latency difference between experiments was larger for the CWS than for the CWNS. This result again is in line with the syntactic defects for the CWS.

## General Discussion

Developmental stuttering is a widely discussed speech fluency disorder. From a psycholinguistic perspective, defects in any type of cognitive processing during speech production can induce disfluency, such as stuttering. An influential model called the EXPLAN model proposes that an atypical interface between planning (PLAN) and execution (EX) processes results in speech disfluencies (Howell, 2002). That is, when the execution of the previous word is complete, the plan for the current word is not yet finished. This problem should be easy to overcome for fluent speakers if they plan ahead for the current word before the execution of the previous one. Thus, a straightforward assumption is that stuttering speakers adopt a smaller scope of speech planning than fluent speakers, inducing more stuttering during speech. However, a defect in lexical selection can also induce the mismatching problem between planning and execution in stuttering speakers. In the current study, the semantic blocking effect in the blocked-cyclic naming paradigm, which was obtained by subtracting the speech onset latencies or error rates in the heterogeneous blocks from the ones in homogeneous blocks, was taken as an index to test the difficulty of lexical selection and the lexical planning scope for CWS in word and phrase production tasks.

If stuttering speakers have defects in word retrieval, it should take longer for them to prepare the word before articulation theoretically. Thus, the speech onset latencies should be longer for CWS than CWNS in word production task, especially in the heterogeneous blocks when there is no confusion of semantic relations. Moreover, if this defect is related to lexical selection, the semantic blocking effect in speech onset latencies should be larger for CWS than for CWNS (Pellowski and Conture, 2005; Hennessey et al., 2008). However, we did not find such evidence in the word production task in the current study. On the contrary, the semantic blocking effect in onset latencies was smaller for stuttering speakers than fluent speakers, though the onset latencies in heterogeneous blocks were statistically equal between stuttering and fluent speakers. These findings in onset latencies were unlikely due to a simple strategy of speed and accuracy trade-off, or stuttering speakers paying more attention on speech production, as discussed previously. The most reasonable explanation for the smaller latency effect of semantic blocking for the CWS is that stuttering speakers adopt a smaller scope of lexical planning in word production. This speculation was confirmed by the same pattern of the semantic blocking effect in the speech onset latencies observed in the phrase production task.

In the phrase production task, the participants were asked to produce a conjoined noun phrase as “N1 and N2” when the N1 was manipulated into homogeneous and heterogeneous blocks; meanwhile, the N2 was always in heterogeneous condition. In such experimental settings, the semantic blocking effect only reflected the lexical planning for the N1. If the CWS adopted a smaller scope of lexical planning in phrase production too, and did not complete the lexical selection for the first noun, a smaller effect of semantic blocking in onset latencies would be observed for the CWS, similarly as the one in word production. The results in phrase production task were consistent with this expectation. Moreover, a comprehensive analysis merging the data of word and phrase production tasks showed a same pattern of semantic blocking effects in onset latencies in these two tasks. This confirmed that the CWS adopted a smaller scope of lexical planning, which was smaller than the first word, than the CWNS in both word and phrase production.

The pattern of the results in error rates was also the same in word and phrase production tasks. First, the CWS were found to consistently make more speech errors than the CWNS. It is not surprising given group definition and classification procedures. Second, a semantic blocking effect of error rates was observed for both CWS and CWNS. This was consistent with our assumption that children's lexical ability was still developing, so they were more vulnerable to the manipulations of semantic blocking than adults. Under the same logic, if the CWS had defects in lexical selection, they would be more vulnerable to the semantic blocking than the CWNS and induce a larger semantic blocking effect in error rates. However, the results turned to be no statistical difference in the magnitude of the semantic blocking effect in error rates between CWS and CWNS. This result confirmed that there is no defect in lexical selection for the CWS.

**Figure 6:**
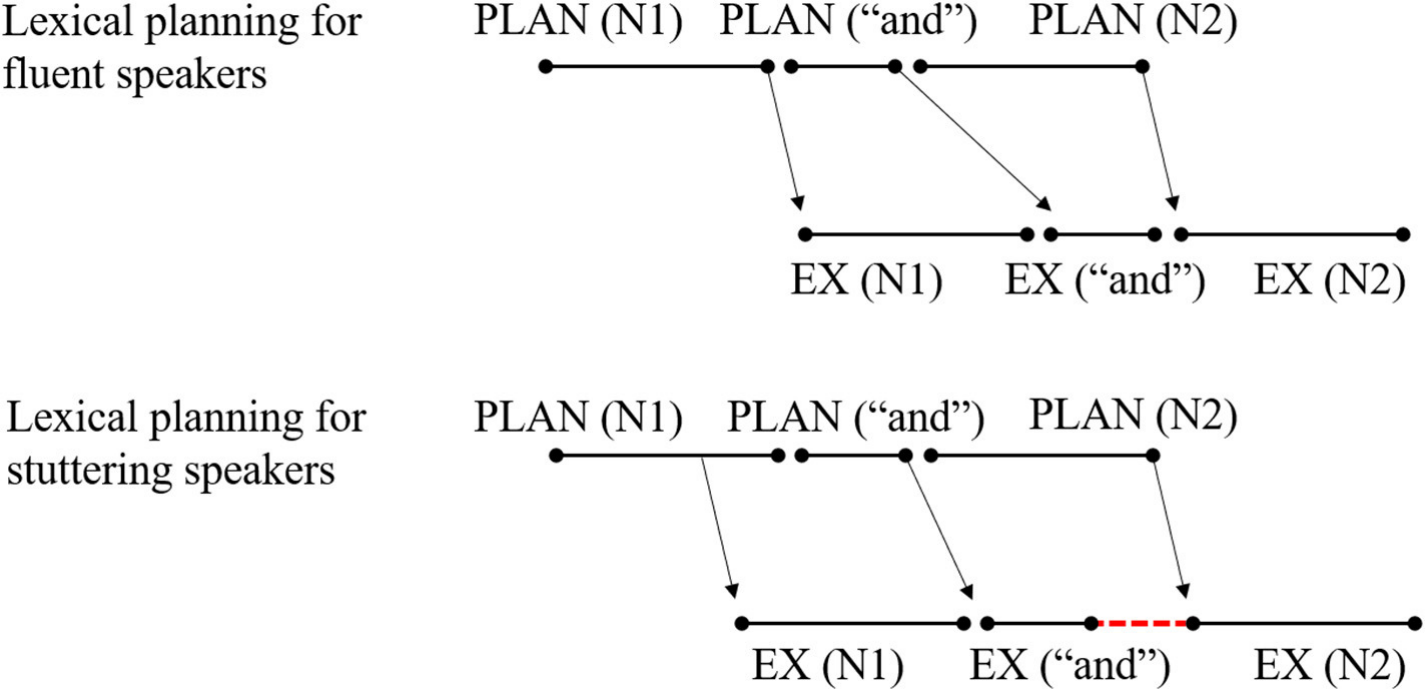
Illustration of lexical planning in conjoined noun phrase production for fluent and stuttering speakers, respectively according to the results of current study.

These results provided more evidence in support of the EXPLAN model. More importantly, they provided further explanation as to why stuttering speakers face the atypical interface between the PLAN and EX processes more frequently. According to the EXPLAN model, both defects in word retrieval and inappropriate planning scope can induce this atypical interface (see Figures 1A,C). The results of the current study distinguished these two origins and supported a smaller planning scope rather than defects in word retrieval as the stuttering mechanism. This was also consistent with the original assumption of the EXPLAN model (Savage and Howell, 2008). In addition, the inappropriate planning scope was specifically related to lexical selection. As shown in Figure 6, when the speakers prepared and produced a conjoined noun phrase, such as “N1 and N2,” fluent speakers completed the lexical selection of the N1 before speech onset (top part), whereas the stuttering speakers adopted a smaller scope of lexical planning (bottom part). Thus, it would be easy for stuttering speakers to make errors on the N1 and face the gap between the execution of “and” and N2. However, this was a simplified example that only considered lexical planning. The exact planning scope of syntactic and phonological encodings for stuttering speakers, and how they adjust them, still need further investigation.

The current study also provided some implication for the syntactic encoding in stuttering. It took more time for the stuttering speakers to initiate the articulation of a phrase, but not of a single word, than the fluent speakers in heterogeneous blocks. Thus, this difference in phrase production is more likely due to syntactic processing than word retrieval. This is also consistent with a previous finding that stuttering speakers have defects in syntactic processing (e.g., Ratner and Sih, 1987; Kleinow and Smith, 2000; Cuadrado and Weber-Fox, 2003). Previous researches showed that the fluent speakers syntactically planned for the conjoined noun phrase before speech onset (Wheeldon et al., 2013; Zhao and Yang, 2016). If the stuttering speakers adopt a similar scope of syntactic encoding, it would take more time for them to initiate the phrase and other longer utterances. Thus, stuttering speakers would have high time pressure to initiate the speech in daily conversation as long utterances are frequently needed. This is consistent with the speculation that CWS go on to persist because of environmental influences, such as the high pressure involved with taking turns speaking (Howell et al., 1999).

It is worth noting that the children who participated in this study were all over 12 years old. Many studies have indicated that most developmental stuttering recovers spontaneously before teenage, even in the early childhood (Yairi and Ambrose, 1999; Ryan, 2001; Howell, 2002), though the severity ratings of the recovered speakers drop dramatically at age 12 and onward (Howell et al., 2008). Thus, the probability of spontaneous recovery for these participants in the future would be very low. As such, it would be better to follow up on their development of stuttering. So far, the results of the current study would be more likely to reflect the properties of persistent stuttering that would continue into adulthood. The smaller scope of lexical planning observed in the current study, a reflection of an atypical interface between planning and execution processes for stuttering speakers, was consistent with imaging data showing the neural bases of atypical planning and execution processes involved in AWS (Lu et al., 2010).

Whether there are defects in word retrieval in stuttering speakers is a topic of heated debate, in both lexical and phonological encoding. First, for lexical encoding, most studies have indicated that a defect can be identified in children prior to the age 6 (Pellowski and Conture, 2005; Hartfield and Conture, 2006). As lexical competence is still developing at that age (Papalia et al., 2007), these types of stuttering may be caused by poor lexical competence and are very likely to be corrected spontaneously with lexical development. Some studies also concluded a lexical defect in AWS, but the literature in this area is limited not only in the number of studies completed but also in the methods used. For example, Bosshardt and Fransen (1996) found that the AWS were slower when monitoring for category-specific words in a silent reading task. It is difficult to distinguish the causes of this effect between the lexical defect and AWS being more vulnerable to interference from concurrent attention-demanding tasks (Bosshardt et al., 2002). However, a study using the classic picture–word interference paradigm in speech production area found no evidence for lexical defects in AWS (Hennessey et al., 2008), which was consistent with our findings.

Finally, for the phonological encoding, an influential model called CRH proposes that stuttering is caused by defects in word retrieval, especially in phonological encoding (Kolk and Postma, 1997). Although we did not test it directly, the non-difference in word production latencies of heterogeneous blocks between stuttering and fluent speakers implied no defects in word retrieval, including both lexical and phonological encoding. Many studies involving overt naming also showed no difference in latencies between AWS and their controls (Sasisekaran et al., 2006; Newman and Bernstein Ratner, 2007). However, these results were considered as evidence for no defects in phonological encoding, as speakers were assumed to complete phonological encoding before a single word is produced, which is still in debate. Some studies have found that the phonological planning scope can be smaller than a lexical word, such as one syllable (Schriefers and Teruel, 1999). Future research is in need to explore whether stuttering speakers also adopt a smaller scope of phonological planning. In addition, evidence for phonological defects in stuttering mainly came from more error rates or stuttering in AWS, which was also consistent with our findings. Another part of this evidence came from findings that AWS were more vulnerable to the syllable- or word-frequency effect (Hubbard and Prins, 1994; Prins and Main, 1997; Newman and Bernstein Ratner, 2007). However, Laganaro and Alario (2006) located the syllable-frequency effect at the phonetic encoding rather than phonological and motor programming levels. Therefore, even if the onset latencies and error rates increase with syllable or word complexity for stuttering speakers, the defects may locate in phonetic rather than phonological encoding. Future studies on this issue need to be more detailed.

In conclusion, we found a smaller scope of lexical planning for stuttering speakers in word and phrase production tasks, which supported the EXPLAN model. These results also provided a detailed explanation for the atypical interface between planning and execution processes for stuttering. In the future, other planning scopes, such as syntactic and phonological encoding should be tested for stuttering speakers. In addition, we did not find evidence for defects in word retrieval, but this also needs further testing.

## Data Availability Statement

The raw data supporting the conclusions of this article will be made available by the authors, without undue reservation.

## Ethics Statement

The studies involving human participants were reviewed and approved by Ethics Committee of the Academy of Psychology and Behavior, Tianjin Normal University. Written informed consent to participate in this study was provided by the participants' legal guardian/next of kin.

## Author Contributions

LZ conceived and designed the experiments, analyzed the data, and wrote the paper. ML performed the experiments. Both authors contributed to the article and approved the submitted version.

## Conflict of Interest

The authors declare that the research was conducted in the absence of any commercial or financial relationships that could be construed as a potential conflict of interest.

## Appendix

**Table:** Stimuli used in the current experiments (the corresponding Chinese pronunciation was presented in slices).

**Category**	**Items**		
Zoo animals	gorilla/xing'xing/	elephant/da'xiang/	zebra/ban'ma/
Fruits	apple/ping'guo/	banana/xiang'jiao/	grapes/pu'tao/
Furniture	dresser/chou'ti/	chair/yi'zi/	couch/sha'fa/
Transports	airplane/fei'ji/	car/qi'che/	boat/chuan/
Musical instruments	drum/gu/	guitar/ji'ta/	piano/gang'qin/
Body parts	ear/er'duo/	finger/shou'zhi/	eye/yan'jing/
